# Dynamic Anti‐Icing Surfaces (DAIS)

**DOI:** 10.1002/advs.202101163

**Published:** 2021-09-09

**Authors:** Feng Wang, Yizhi Zhuo, Zhiwei He, Senbo Xiao, Jianying He, Zhiliang Zhang

**Affiliations:** ^1^ NTNU Nanomechanical Lab Department of Structural Engineering Norwegian University of Science and Technology (NTNU) Trondheim 7491 Norway; ^2^ College of Materials and Environmental Engineering Hangzhou Dianzi University Hangzhou 310018 China

**Keywords:** dynamic anti‐icing surfaces, icephobic materials, low ice adhesion, surface icephobicity

## Abstract

Remarkable progress has been made in surface icephobicity in the recent years. The mainstream standpoint of the reported antiicing surfaces yet only considers the ice–substrate interface and its adjacent regions being of static nature. In reality, the local structures and the overall properties of ice–substrate interfaces evolve with time, temperature and various external stimuli. Understanding the dynamic properties of the icing interface is crucial for shedding new light on the design of new anti‐icing surfaces to meet challenges of harsh conditions including extremely low temperature and/or long working time. This article surveys the state‐of‐the‐art anti‐icing surfaces and dissects their dynamic changes of the chemical/physical states at icing interface. According to the focused critical ice–substrate contacting locations, namely the most important ice–substrate interface and the adjacent regions in the substrate and in the ice, the available anti‐icing surfaces are for the first time re‐assessed by taking the dynamic evolution into account. Subsequently, the recent works in the preparation of dynamic anti‐icing surfaces (DAIS) that consider time‐evolving properties, with their potentials in practical applications, and the challenges confronted are summarized and discussed, aiming for providing a thorough review of the promising concept of DAIS for guiding the future icephobic materials designs.

## Graphical abstract

Combating excessive icing through designing and deploying material surfaces has received growing interests. In this review, the practical design principles of anti‐icing surfaces through enabling dynamic changes in the chemical/physical states of the ice/substrate/ice–substrate interface with tailored functions by the three dynamic anti‐icing surfaces categories, namely, surfaces with dynamic substrates, dynamic interfaces, and dynamic ice structure are summarized.

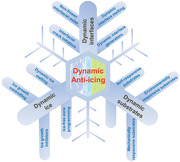

## 1 Introduction

Icing is one of the most common natural phenomena that greatly impact human activities. Undesired ice formation and accumulation can result in numerous safety problems to aircraft, power grid, transmission line, roadway, marine vessel, renewable energy infrastructure, and many others.^[^ ^1^, ^2^, ^3^, ^4^, ^5^, ^6^, ^7^, ^8^ ^]^ Traditional methods used for dealing with icing problems, for instance, mechanical deicing, thermal or chemical treatments, are often highly costly and at the same time low‐efficient.^[^ ^9^, ^10^ ^]^ As such, enormous interests have been aroused in deploying surfaces that can control icing and mitigate its related damages. The so‐called icephobic or anti‐icing surfaces with properties like repelling incoming water droplets, delaying ice nucleation, repressing ice growth and weakening ice adhesion are designed for anti‐icing purpose.^[^ ^11^, ^12^, ^13^, ^14^ ^]^ From the early lotus‐leaf inspired superhydrophobic surfaces (SHS) fabricated for repelling water droplets and delaying ice nucleation to the recent omniphobic pitcher‐plants‐inspired slippery liquid‐infused porous surfaces (SLIPS) developed for multiple anti‐icing,^[^ ^11^, ^12^, ^15^, ^16^, ^17^, ^18^, ^19^, ^20^, ^21^, ^22^, ^23^, ^24^ ^]^ there are currently a colorful spectrum of anti‐icing surfaces reported in the literature showing great potentials with practical low ice adhesion strength 0.2–10 kPa (generally, icephobic surfaces are defined as *τ*_ice_ < 100 kPa, and the passive removal of ice requires much lower value *τ*_ice_ < 10 kPa) and easy achievable large‐scale ice remove capacity.^[^ ^14^, ^25^, ^26^, ^27^ ^]^

Despite the remarkable progress already made in surface icephobicity in the recent years, the anti‐icing surfaces are generally designed from a static perspective, for instance, texturing the surfaces structures, tuning the modulus of substrates, and modifying the surfaces energy without considering the evolution of properties.^[^ ^11^, ^12^, ^13^, ^14^, ^15^, ^16^, ^17^, ^18^, ^19^, ^20^, ^21^, ^22^, ^23^, ^24^ ^]^ One of the key characteristics of these surfaces is that the ice–substrate contact area after ice formation is regarded as “constant.” The main focus of surface design lies on the states before ice formation but not the dynamic changes after icing. For instance, the anti‐icing performance of SHS including repelling incoming water and delaying ice nucleation has been widely discussed,^[^ ^11^, ^12^, ^17^, ^18^, ^19^ ^]^ with attentions only on the phenomena before ice formation. However, once ice forms on surfaces, especially in icing/deicing cycles, the anti‐icing performance of the SHS decays very fast because the surfaces asperities can be easily damaged.^[^ ^18^ ^]^ Another commonly used anti‐icing surface SLIPS with lubricant layer atop that enable the surfaces with excellent repellence to any immiscible materials also focus on the surface activities before icing. After ice formation on SLIPS, the lubricants can be exhausted easily due to ice removal.^[^ ^24^ ^]^ Generally, the static anti‐icing surfaces (SAIS) have disadvantages, for example, inapplicability at extremely low temperature, fragility to surfaces damage and surfaces degradation, and inadaptability to environmental changes.^[^ ^16^, ^17^, ^18^, ^28^, ^29^, ^30^, ^31^, ^32^, ^33^ ^]^

Very recently, we witness a shift in anti‐icing surface design principles from being of static nature, namely, from no consideration on the changes at the ice–substrate contacting areas after ice formation, to the focus on enabling dynamic changes of the chemical/physical states of the ice/substrate/ice–substrate interface for enhanced anti‐icing performances.^[^ ^34^, ^35^, ^36^, ^37^, ^38^, ^39^, ^40^ ^]^ These emerging anti‐icing surfaces can be regarded as dynamic anti‐icing surfaces (DAIS), thanks to the integrated evolving properties that can mitigate the interactions between ice and substrate even after ice formation. Extraordinarily, certain effective approaches in the traditional active deicing methodologies were featured in the design of DAIS. Unlike the direct and abrupt application of external mechanical, chemical, and electrical energy as driving force to remove ice, DAIS integrate the active strategies into the evolution of the ice–substrate contacting regions to reduce the resistance of removing ice. For instance, integrating dynamic antifreeze‐secreting capacity into superhydrophobic surfaces to introduce continuous assistance for ice removal even after ice formation.^[^ ^34^ ^]^ This strategy inherits the advantages of superhydrophobic surfaces in preventing ice forming and triggers evolution of properties at icing region after ice formation for easy ice removal. New anti‐icing surfaces with photothermal traps can actively melt interfacial ice, which utilize solar energy in deicing directly.^[^ ^38^ ^]^ Importantly, DAIS exhibit improved durability, wider temperature tolerability, and better environment adaptivity, and thus have gained increasing interests in the anti‐icing research and application fields.^[^ ^14^, ^35^, ^36^, ^37^, ^38^, ^39^, ^40^ ^]^

**Figure 1:**
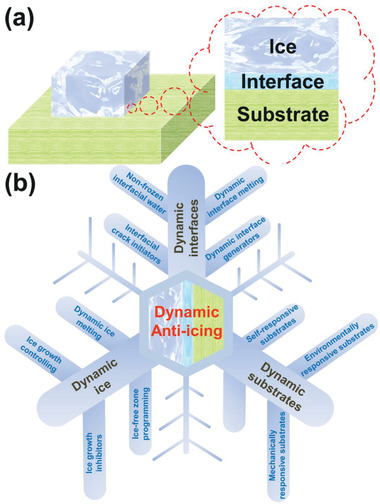
DAIS. a) The three most important regions close to the ice–substrate interface determine anti‐icing performance of a surface. b) DAIS targeting the three ice–substrate interfacial regions. The dynamic substrates include substrates that can respond to the internal/external conditions, namely those by tuning the surface state and affecting the ice formation/adhesion on the top. The dynamic interfaces cover the surfaces that can introduce dynamic evolution in the chemical/physical states of the ice–substrate interface after ice formation, thus facilitating easy ice removal. The dynamic ice encompasses the surfaces that can tailor ice growth, propagation or even melt ice for the purpose of mitigating ice accumulation.

This review aims to provide a thorough survey on the newest development of DAIS. Focusing on the most relevant ice–substrate interfacial regions (**Figure** **1**a), and their spontaneous/stimuli‐responsive changes in the chemical/physical states impacting ice adhesion during and after ice formation. The state‐of‐the‐art anti‐icing surfaces are reassessed and classified into three categories, namely, surfaces with dynamic substrate, dynamic interface, and dynamic ice changes, as shown in Figure 1b. Surfaces with dynamic properties in the substrate generally include functional structures that respond to internal and external stimuli, and thus modify the substrate properties and enhance anti‐icing performances.^[^ ^36^, ^37^, ^41^, ^42^, ^43^, ^44^ ^]^ Surfaces with dynamic properties at the ice–substrate interfaces provide the possibility of altering interface interactions for lowering ice adhesion.^[^ ^14^, ^35^, ^39^, ^40^, ^45^, ^46^, ^47^ ^]^ Surfaces with dynamic ice are able to direct ice growth, propagation, and even ice melting, which can mitigate ice accumulation and assist ice removal on the surfaces.^[^ ^13^, ^38^, ^48^, ^49^, ^50^, ^51^, ^52^, ^53^, ^54^ ^]^ One can see that DAIS is introduced in this review with the purpose of promoting rethinking of the design principles of new anti‐icing surfaces rather than recategorizing of the known anti‐icing materials. It should be emphasized that the traditional deicing methodologies are still actively in use despite their obvious shortcomings. Under the DAIS concept, the focus is to utilize the evolution of the ice–substrates and its adjacent region in materials design, but not to distinguish active or passive deicing. The following sections detailed the categories of DAIS surfaces.

## 2 Dynamic Substrates

### 2.1 Self‐Responsive Substrates

**Figure 2:**
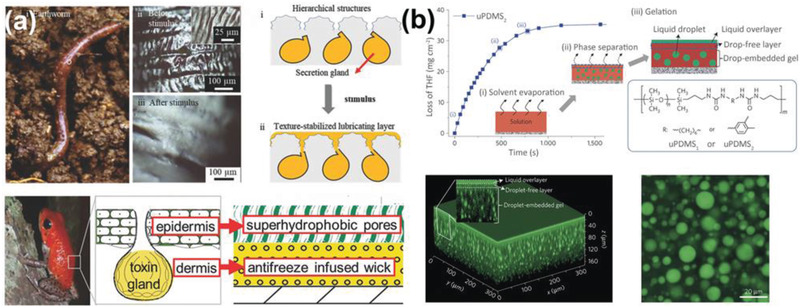
DAIS with self‐responsive substrates. a) The lubricant regenerable systems in earthworm and poison dart frog, and the inspired self‐responsive substrates design. Upper part: Adapted with permission.^[^ ^55^ ^]^ Copyright 2018, Wiley‐VCH. Lower part: Adapted with permission.^[^ ^34^ ^]^ Copyright 2015, Wiley‐VCH. b) The droplet‐embedded gel structure, and its secreted surface lubricating film. Confocal fluorescence images of the substrates visualized the lubricant film on the substrate surface and the internal distribution of the lubricant droplets. Adapted with permission.^[^ ^56^ ^]^ Copyright 2015, Nature Publishing Group.

Many surfaces exhibit dynamic changes in response to their internal forces. Such self‐responsive surfaces are widely observed in natural organisms and systems. For instance, as shown in **Figure** **2**a, earthworms and poison dart frogs have secretion glands under their skin, which release lubricant to form a slippery layer above the skin.^[^ ^34^, ^55^ ^]^ Such surface lubricating is driven by the under‐skin disjoining pressure or concentration gradient.^[^ ^42^, ^56^ ^]^ The mechanism underling self‐responsive surfaces has inspired the design of anti‐icing surfaces with embedded lubricants in the substrates.

One notable self‐responsive substrate was developed through phase separation, as shown in Figure 2b. By first dissolving urea and polydimethylsiloxane (uPDMS) copolymers and excess silicon oil into tetrahydrofuran (THF) and then the subsequent evaporation of THF, the resulting crosslinked polymer matrix trapped the excessive silicone oil as internal droplets.^[^ ^56^ ^]^ Such droplet‐embedded gel system by phase separation of the microscale oil droplet nucleation and formation was metastable, and was able to secrete a lubricating film on the surface with a spreading factor of *S* = *γ*_ga_ – (*γ*_la_ – *γ*_gl_) > 0, where *γ*_ga_, *γ*_la_, and *γ*_gl_ are the gel–air, liquid–air, and gel–liquid interfacial tensions, respectively.^[^ ^57^ ^]^ The surface lubricant layer was regenerable under the driving force of disjoining pressure originated from the van der Waals interactions at the gel surface.^[^ ^56^ ^]^ Both the thickness of the lubricant layer and the size of the embedded droplets in this substrate were controllable through polymer crosslinking strength and oil content, which offered a strategy for preparing similar substrates for improving anti‐icing performance.^[^ ^34^, ^41^, ^42^, ^43^, ^58^, ^59^, ^60^, ^61^, ^62^, ^63^, ^64^, ^65^, ^66^, ^67^ ^]^ Icephobic surfaces following the same strategy for surface regenerable lubricant layers indeed showed ice adhesion strength below 40 kPa,^[^ ^41^ ^]^ with regenerated lubricating layers after 15 wiping/regenerating tests and long‐term ice adhesion strength below 70 kPa. Through a precise proportioning of polymer and oil, novel self‐lubricating organogels (SLUG) displayed extremely low ice adhesion strength of 0.4 kPa.^[^ ^43^ ^]^ The ice formed on the SLUG with small tilting angles could slide off at −15 °C, which demonstrated the great potential of self‐responsive substrates in anti‐icing.

**Figure 3:**
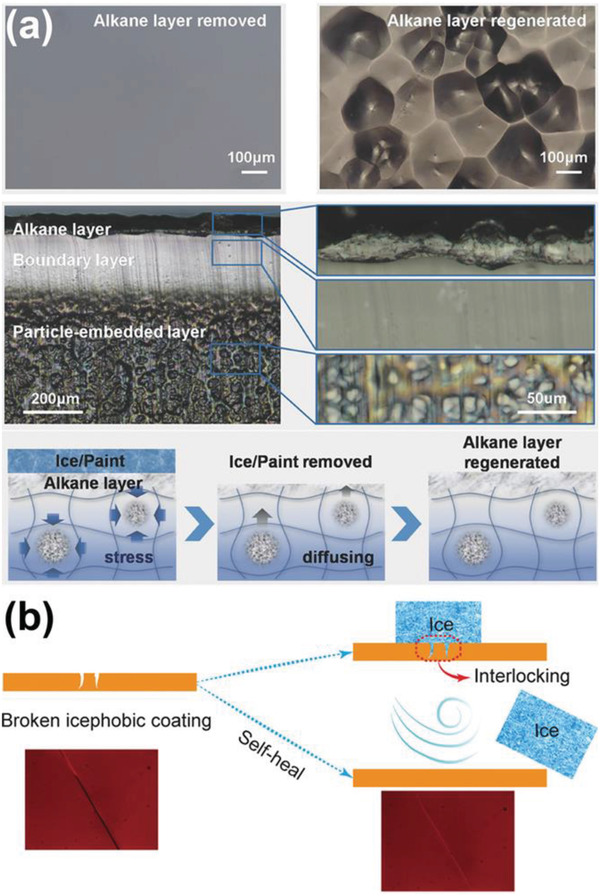
DAIS with self‐responsive substrates. a) The alkane‐embedded structure, showing the alkane distribution in the substrate, and the regeneration mechanism of the solid alkane layer. Adapted with permission.^[^ ^42^ ^]^ Copyright 2017, Wiley‐VCH. b) The schematic of the self‐healing icephobic surface. Adapted with permission.^[^ ^37^ ^]^ Copyright 2018, American Chemical Society.

Besides small liquid oil molecules, solid lubricants were also used in self‐responsive substrates for regenerating surface lubricating layers to facilitate ice removal.^[^ ^42^, ^61^ ^]^ For example, alkane embedded in a polymer substrate can diffuse onto the surface driven by the concentration gradient and the internal stress of the polymer matrix, resulting in a solid alkane layer as shown in **Figure** **3**a. The regenerable solid lubricant alkane had weak interactions with the polymer substrate and served as a sacrificial layer in ice removal, which enabled low ice adhesion strength (≈9 kPa) and good durability in 20 icing/deicing cycles.^[^ ^42^ ^]^ Recently, perfluoroalkane wax was verified as a good lubricant candidate for fabricating solid‐lubricant regenerable surfaces.^[^ ^61^ ^]^ The perfluoroalkane wax regenerating surface demonstrated low ice adhesion strength (≈20 kPa) and high environmental stability. Comparing to the liquid counterpart, solid lubricant layers are mechanically more robust in icing/deicing cycles. For both liquid and solid lubricant regenerable surfaces, the limited regenerating capacity of surfaces hinders their application. Unlike earthworm and poison dart frog that can secrete lubricant from body, the embedded lubricant in the surfaces can be used out easily. Therefore, future studies need to focus on self‐responsible surfaces with long‐term lubricant regenerating capacity.

Surface damages in practical applications are severely harmful to the icephobicity and durability of anti‐icing surfaces, since any possible interlocking between ice and surface can greatly enhance ice adhesion. Therefore, self‐responsive substrates with self‐healing functionality have been fabricated for anti‐icing purposes.^[^ ^36^, ^37^, ^58^, ^68^, ^69^, ^70^, ^71^ ^]^ Such substrates showed significant improvements in mechanical durability because of the self‐healing ability of surface damages at subzero temperature to maintain their smooth topographies (Figure 3b). One of the self‐healing substrates, Fe‐pyridinedicarboxamide‐containing PDMS (FePy‐PDMS) elastomer, exhibited low ice adhesion strength of ≈6 kPa after 50 icing/deicing cycles.^[^ ^37^ ^]^ In order to increase the self‐healing rate during icing/deicing cycles, ultrafast self‐healing and highly transparent (the coated glass showed light transmittance of 89.1%) icephobic substrates were prepared by optimizing polymer chain flexibility and concentration of hydrogen bonding, which can restore ≈80% of the ultimate tensile strength of the substrate after healing for 45 min at room temperature.^[^ ^72^ ^]^ The self‐healing substrates can be promising candidates for robust anti‐icing surfaces in practical environment if the self‐healing functionality is further improved especially at low temperature.

### 2.2 Environmentally Responsive Substrates

**Figure 4:**
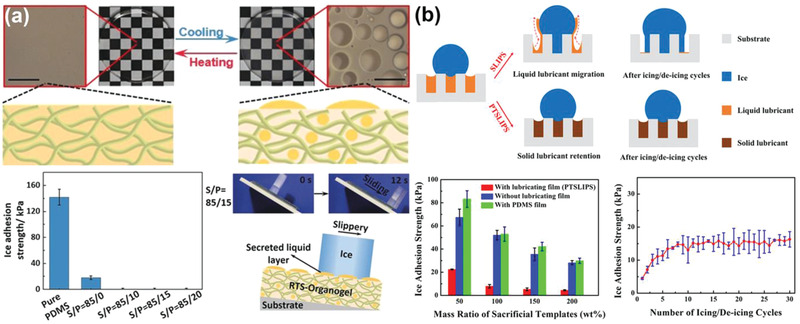
DAIS through environmentally responsive substrates. a) The thermal responsive lubricant regenerable organogel, showing the droplets secretion at low temperature and reversible droplets absorption at high temperature. Low ice adhesion enabled small sliding angle of ice on the organogel at −15 °C was showed with schematics with corresponding samples (the S/P means the mass ratio of silicone oil and liquid paraffin). Adapted with permission.^[^ ^44^ ^]^ Copyright 2020, Wiley‐VCH. b) The phase transformable slippery liquid infused porous surfaces, showing enhanced lubricant durability comparing to slippery liquid infused porous surfaces. Adapted with permission.^[^ ^36^ ^]^ Copyright 2019, Elsevier Publishing Group.

Many anti‐icing surfaces use dynamic substrates that respond to the ambient conditions of temperature, magnetic field, light, and so on.^[^ ^36^, ^44^, ^73^, ^74^, ^75^, ^76^, ^77^, ^78^, ^79^, ^80^, ^81^ ^]^ By integrating temperature sensitive components during fabrication, anti‐icing substrates can respond to temperature change in the surrounding.^[^ ^44^, ^73^ ^]^ One of such substrates incorporated a binary liquid mixture (silicon oil and liquid paraffin) into a PDMS network, and the resulting reversibly thermosecreting organogel (RTS‐organogel) demonstrated distinct morphologies at different temperature, as shown in **Figure** **4**a.^[^ ^44^ ^]^ Specifically, the substrate was transparent at high temperature as the internal silicon oil and liquid paraffin were miscible. Upon cooling to icing temperature, the substrate secreted visible oil droplet to its surface owing to the collapse of the polymer matrix and the phase separation of the silicon oil from paraffin. Unlike the liquid layer propelled by internal force in the above‐mentioned SLUG shown in Figure 2b, the oil film generated on this substrate was largely driven by temperature. Nevertheless, the lubricating oil film secreted at low temperature was notably beneficial for the icephobicity of the surfaces. The ice adhesion strength on the RTS‐organogel was less than 1 kPa at −15 °C, which enabled sliding of ice cube on the surface samples with small tilting angles.^[^ ^44^ ^]^ The RTS‐organogel was believed to be more durable than the SLUG, because they could reversibly absorb lubricant into the polymer matrix for replenishing the internal lubricants in storage and against evaporation/contamination.

Liquid lubricants in anti‐icing substrates can be depleted in limited number of icing/deicing cycles due to the weak interaction between the lubricant and the base materials, which leads to poor durability.^[^ ^32^ ^]^ Interestingly, special temperature responsive lubricants provide a feasible solution to avoid such issue. As shown in Figure 4b, a phase transformable lubricant was used in creating the so‐called phase transformable slippery liquid infused porous surfaces (PTSLIPS) recently.^[^ ^36^ ^]^ Because the lubricant had a phase transition point from liquid to solid above water freezing point at ≈3 °C,^[^ ^36^ ^]^ the durability of the fabricated PTSLIPS was greatly improved. The PTSLIPS showed a low ice adhesion strength of ≈4 kPa and long‐term ice adhesion strength of 16 kPa after 30 icing/deicing cycles. Although the PTSLIPS can be fabricated using daily accessible materials like foams and common paper, its anti‐icing performance was superior than the results obtained on other conventional SLIPS used for anti‐icing. Because the phase transition point of the lubricant in the PTSLIPS was only marginally above water freezing temperature, displaced lubricants on the substrate surface in deicing can be restored in the subsequent phase transition cycle of the lubricant at slightly increased temperature, rendering self‐healing abilities and promising application potentials. Furthermore, the solid lubricant can physically prevent water from permeating into the substrate, thus suppressing mechanical interlockings during ice formation on the substrate. It is worth noting here that similar phase change materials (PCMs) were introduced into concrete for anti‐icing. During the phase transition upon cooling, the PCMs released substantial latent heat and hindered ice accumulation in walking pavements.^[^ ^74^, ^75^, ^76^, ^82^, ^83^, ^84^ ^]^ Although the RTS‐organogel and PTSLIPS significantly increase the durability of anti‐icing surfaces, the soft polymer matrices limit their mechanical robustness. Matrices with superior mechanical and chemical resistance should be explored for further improving their durability.

**Figure 5:**
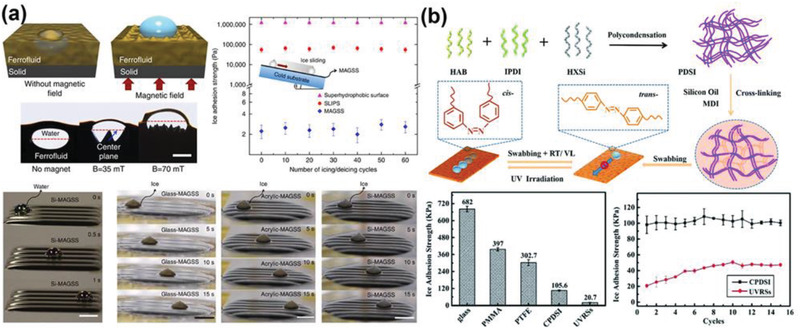
DAIS through environmentally responsive substrates. a) The magnetic slippery icephobic surfaces, showing stable water/ice repellency with extremely low adhesion under magnetic field. Adapted with permission.^[^ ^77^ ^]^ Copyright 2016, Nature Publishing Group. b) The UV‐responsible substrates, showing UV irradiation controlled lubricant release and low ice adhesion strength. Adapted with permission.^[^ ^79^ ^]^ Copyright 2020, Royal Society of Chemistry.

Magnetic field is currently not often applied in anti‐icing practice. However, utilizing magnetic field as stimuli to modulate substrates has been reported, with encouraging results illustrating the potentials in active deicing technology.^[^ ^77^, ^78^, ^80^ ^]^ The magnetic slippery surfaces (MAGSS) were the successful representatives of this type of anti‐icing substrates, which used magnetic fluid (that is, ferrofluid) along with a magnetic field for controlling surface properties, as shown in **Figure** **5**a.^[^ ^77^ ^]^ The MAGSS responded to external magnetic field and generated a volumetric force to suppress water droplets from sinking into the substrate body with bulk oil, which led to small water droplet sliding off angle of 2.5°. Remarkably, the MAGSS maintained its liquid‐like phase at low temperature and were highly slippery to ice, showing extremely low ice adhesion strength (≈2 Pa) without degradation after 60 icing/deicing cycles.

Light is another important stimulus source for triggering dynamic changes in anti‐icing substrates.^[^ ^85^, ^86^, ^87^, ^88^ ^]^ By integrating light‐absorbing azobenzene groups into polymer skeleton of the base materials, the so‐called UV responsive substrates (UVRS) were able to utilize UV energy for polymer chain conformation conversion.^[^ ^79^ ^]^ As shown in Figure 5b, the integrated azobenzene groups in the UVRS changed from trans‐ to cis‐conformation under UV light with a wavelength of 365 nm, resulting in slight compression of the whole substrate. The pre‐embedded silicon oil in the polymer matrix of the UVRS was released to the substrate surface as a response to the compressive stress, which enabled low ice adhesion strength of 21 kPa and long‐termed ice adhesion strength of 47 kPa after 15 icing/deicing cycles on the UVRS. The light stimuli also had thermal effects, which further inspired the design of photothermal responsive substrates. Cocoa oil with low‐melting point and efficient photothermal Fe_3_O_4_ nanoparticles were infused into porous structures together in fabricating lubricant‐infused surfaces (LIS).^[^ ^81^ ^]^ Upon irradiation with infrared light, the LIS can absorb optical energy via Fe_3_O_4_ nanoparticles, and led to melting of the internal solid cocoa oil as lubricant for surface slippery liquid. The LIS thus possessed switchable hydrophobic/slippery functionality by turning off/on the light source. The smart responses of substrates to magnetic field, UV light and infrared light enhance icephobicity of the surfaces, however, and limit the application scopes of these surfaces at the same time. Novel environmentally responsive substrates which can function under more universal conditions are in demand.

### 2.3 Mechanically Responsive Substrates

**Figure 6:**
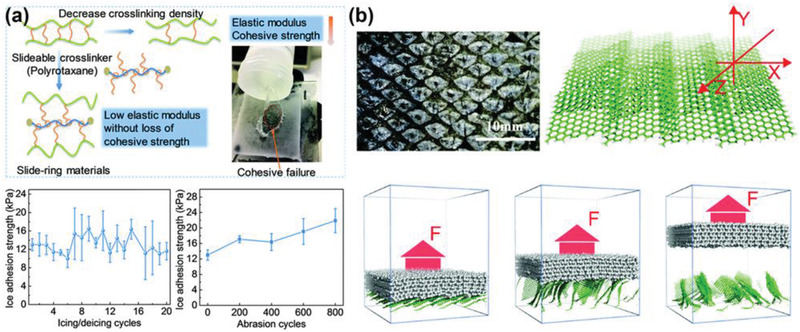
DAIS through mechanically responsive substrates. a) The slide‐ring substrate, showing its molecular mechanism and the enhanced cohesive strength and excellent durability. Adapted with permission.^[^ ^89^ ^]^ Copyright 2019, Royal Society of Chemistry. b) The fish‐scale‐like surface, showing sequential rupture of atomistic interactions for lowering atomistic ice adhesion. Adapted with permission.^[^ ^90^ ^]^ Copyright 2019, Royal Society of Chemistry.

Ice removal generally involves stress change on the ice adhered surface. Mechanically responsive substrates utilize the surface structures that can be dynamically altered under stress to achieve low ice adhesion. The surface structures that respond to the mechanical force can be both molecular structures in base materials and/or geometrical patterns above the surfaces.^[^ ^89^, ^90^ ^]^ Notably, an ultradurable icephobic coating was designed by introducing slide‐ring crosslinkers, namely, molecular pulleys, into PDMS base matrix (**Figure** **6**a).^[^ ^89^ ^]^ The slide‐ring crosslinkers were able to not only move along the polymer chains under a mechanical loading, but also return to their original state via entropic repulsion upon relieving loading.^[^ ^91^, ^92^ ^]^ The unique structures in this substrate decreased the elastic modulus of the polymer matrix for low ice adhesion, and at the same time guaranteed excellent cohesive strength for mechanical durability, mitigating the common problem of other low elastic modulus polymer materials used for anti‐icing. The slide‐ring substrate showed a low ice adhesion of ≈12 kPa during 20 icing/deicing cycles, maintained ice adhesion strength ≈22 kPa after 800 abrasion cycles, and become one of the most durable elastomers reported so far.

In another report, an interesting fish‐scale‐like dynamic anti‐icing surface prototype was introduced (Figure 6b).^[^ ^90^ ^]^ By taking a deep look into sequential and concurrent ice detaching modes from substrates, atomistic modeling, and molecular dynamics simulations were utilized to reveal mechanisms for lowering ice adhesion at the atomistic interaction level. Because the atomistic interactions ruptured all at once in concurrent rupture mode but incrementally in the sequential rupture mode, opening of the surfaces structure featuring the sequential rupture of ice was essential for low ice adhesion. Inspired by the structure topology of fish scales, the fish‐scale‐like surface was designed by piling of graphene platelets in atomistic modeling. Under deicing forces, the graphene platelets dynamically opened up to enable sequential rupture of ice from the surface, which led to a ≈60% reduction in ice adhesion strength. The theoretical model of fish‐scale‐like surface was a good starting point of mechanically responsive structure design for low ice adhesion. Experimental fabrication of fish‐scale‐like dynamic anti‐icing surfaces is needed for the validation of the modeling results and further to trigger realistic applications.

## 3 Dynamic Interfaces

### 3.1 Nonfrozen Interfacial Water

**Figure 7:**
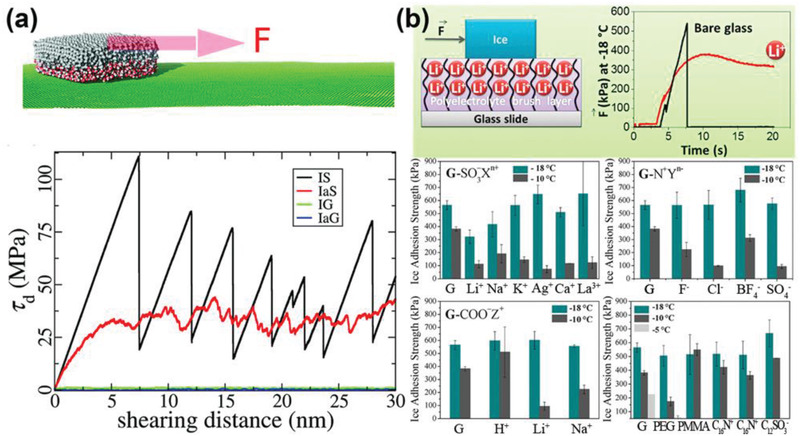
DAIS with nonfrozen interfacial water layer. a) Atomistic modeling and simulation of ice adhesion on different surfaces with/without an interfacial aqueous water layer. The ice cube directly sitting on the silicon surface, ice cube with a sandwiched water layer on the silicon surface, ice cube on graphene, and ice cube with sandwiched water on graphene were named as IS, IaS, IG, and IaG, respectively. Adapted with permission.^[^ ^101^ ^]^ Copyright 2016, Royal Society of Chemistry. b) The ice adhesion on the bare glass, G, and on polyelectrolyte brush layers comprising of different types of counterions (X*^n^* ^+^ = Li^+^, Na^+^, K^+^, Ag^+^, Ca^2+^, C_16_N^+^, La^3+^; Y*^n^* ^−^ = F^−^, Cl^−^, BF_4_^−^, C_12_SO_3_^−^, SO_4_^2−^; and Z^+^ = H^+^, Li^+^, C_16_N^+^, Na^+^). Adapted with permission.^[^ ^108^ ^]^ Copyright 2014, American Chemical Society.

The importance of the ice–substrate interface to ice adhesion is self‐evident. It is known that ice is slippery to ice‐skating blades, meaning low adhesion strength, due to a surface premelted layer.^[^ ^93^ ^]^ It is known that there is a premelted liquid or liquid‐like aqueous layer that exists on the surface of ice at subzero temperature because of regelation or by pressure or friction melting.^[^ ^93^, ^94^, ^95^, ^96^, ^97^ ^]^ The premelted or liquid‐like layer of ice can be observed not only at ice–vapor but also ice–solid interfaces.^[^ ^98^, ^99^ ^]^ As the thickness of the premelted layer at ice–solid interfaces was found to increase with temperature, utilizing and amplifying the premelted layer for effectively reducing ice adhesion became one of the important strategies of surface icephobicity.^[^ ^100^ ^]^ Because the premelted layers have thickness at the nanoscale, atomistic modeling, and molecular dynamic simulation were employed to investigate their effects on the nanoscale ice adhesion.^[^ ^101^ ^]^ As shown in **Figure** **7**a, ice‐cube models with premelted interfacial water layers on solid substrates showed negligible ice adhesion stress if compared with ice directly contacted with the solid substrate. The premelted layer converted ice–substrate contact from a solid–solid to a solid–liquid–solid manner, which resulted in weak interfaces with disordered and short‐term atomistic interactions of van der Waals forces and hydrogen bonds. Related studies focused on the lubricating effect of the premelted or nonfrozen interfacial water layer further supported the potential of utilizing aqueous layer for mitigating icing problems.^[^ ^101^, ^102^, ^103^ ^]^

Although interfacial nonfrozen water layers were identified at the ice–solid contact interfaces as early as in 2004, further experimental explorations on their application in anti‐icing came much later.^[^ ^99^, ^100^ ^]^ Through rational nanostructuring of solid surfaces for creating an interfacial quasi‐liquid layer, ice formation was delayed for 25 h at −21 °C.^[^ ^104^ ^]^ Yet, ice formation is inevitable with given long enough icing time. As ice adhesion on certain solid surfaces, such as SiO_2_, Si, Au, and so on, are too high to any ice removal approach,^[^ ^105^, ^106^ ^]^ new strategies that could program nonfrozen interfacial water on such surfaces for low ice adhesion are in great needs. Using highly hydrated ions was believed to be a good approach for creating quasi‐liquid layers (QLLs) on solid surfaces, because ions can greatly impact the structure of water and suppress ice nucleation.^[^ ^107^ ^]^ Polyelectrolyte brushes hosting ions was employed to probe the effects of counter ions on ice adhesion (Figure 7b).^[^ ^108^ ^]^ It was found that the polyelectrolyte brushes with kosmotropic counterions (G‐SO^3−^Li^+^, G‐SO^3−^Na^+^) had maximum ice adhesion reduction (25–40%) comparing to the bare glass (G) at −18 °C, because of the most negative water structural entropy resulting from strong hydration. In comparison, the polyelectrolyte brushes with chaotropic counterions (G‐SO_3_^−^ K^+^, G‐N^+^Cl^−^, and G‐N^+^SO_4_^2−^) did not change the ice adhesion, owing to positive water structural entropy of weak hydration.^[^ ^107^ ^]^ The nature of the polyelectrolyte layer also affected ice adhesion significantly. For instance, brushes with G‐SO_3_^−^Li^+^ decreased ice adhesion dramatically, which was not observed on samples with G‐COO^−^Li^+^. Such results suggested that kosmotropic counterions incorporated with strongly dissociating polyelectrolyte brush could facilitate ice removal. This study provided important reference for integrating counterions for related anti‐icing applications.^[^ ^109^ ^,[^ ^110^ ^]^

**Figure 8:**
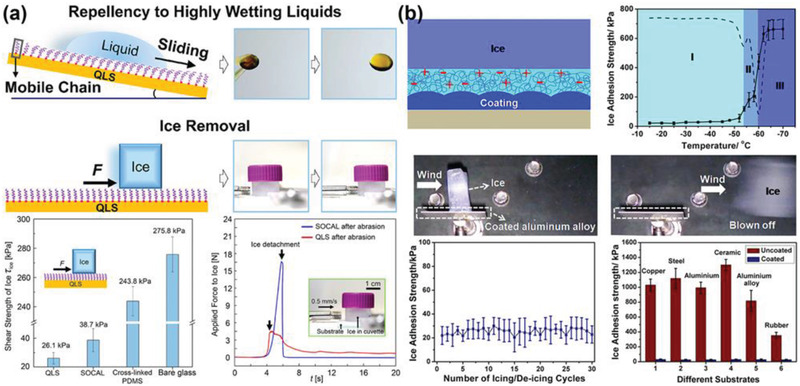
DAIS with nonfrozen interfacial water layer. a) The liquid repellency and ice removal property of the quasi‐liquid surface (QLS). Adapted with permission.^[^ ^114^ ^]^ Copyright 2020, American Chemical Society. b) The reduction of the ice adhesion strength by an aqueous lubricating layer and the anti‐icing performance of the coatings. Adapted with permission.^[^ ^105^ ^]^ Copyright 2014, American Chemical Society.

To improve the lubricant effects for extremely low ice adhesion, the thickness of QLL needs to be drastically increased. Generally, developing liquid‐like surfaces through covalently grafting flexible polymers on flat surfaces was an effective approach. The state‐of‐the‐art liquid‐like surfaces, for example, the slippery omniphobic covalently attached liquid surfaces, still exhibited limited thickness of QLL, which restricted sustainable ice removal.^[^ ^111^, ^112^, ^113^ ^]^ Recently, a nonsticky and extremely flexible quasi‐liquid surface (QLS) with a coating thickness of 30.1 nm was reported (**Figure** **8**a),^[^ ^114^ ^]^ which enabled extreme flexibility and quasi‐liquid thickness of the surface. The QLS had omniphobic nature of exceptional repellency to water and organic liquids and showed extremely low glass transition temperature (*T*_g_ = −125 °C). All the properties enabled low ice adhesion strength of ≈26 kPa on QLS, and deicing possibility by air flows (mimicking wind power).

Although the surfaces with quasi‐liquid layers are highly favourable for lowering ice adhesion strength, maintaining the durability of the grafted polymer chains on such surfaces exposed to mechanical damages is still challenging. Another difficulty of applying quasi‐liquid layers emerges when surfaces roughness scale is larger than the layer thickness. To address these two problems, a robust and durable anti‐icing coating fabricated by polymers (polyurethane, PU) with hydrophilic pendant groups was newly prepared.^[^ ^105^ ^]^ The hydrophilic component dimethylolpropionic acid (DMPA) in the coating could absorb water directly from humid environments or from the contacted ice/snow due to the ion effect (Figure 8b), which resulted in aqueous lubricating layers on the coating surface as long as ice formed. Surface samples the coating containing 9 wt% DMPA (PU‐9) can maintain stable and low ice adhesion strength of 27 kPa at temperature as low as −53 °C. The PU‐9 on the coating also exhibited excellent durability during icing/deicing test, showing almost no change after 30 cycles. It was believed that the coating was adaptable on almost all surfaces including rough ones (Figure 8b, right bottom). The fabrication strategy of the coatings, incorporating hydrophilic pendant groups in soft polymer for aqueous lubricating layer, was thus widely employed in similar studies.^[^ ^39^, ^47^, ^106^, ^115^, ^116^, ^117^, ^118^, ^119^, ^120^ ^]^ Despite the great progresses made on programing QLL at interface for assisting ice removal, ice adhesion strength on these surfaces (≈20 kPa) are still beyond the super low ice adhesion threshold (10 kPa) required for ice self‐removal by its own gravitational force. Further development of surfaces with nonfrozen interfacial water layer will need to target lower ice adhesion strength.

### 3.2 Dynamic Interface Melting

The thickness of interfacial aqueous lubricant layers introduced above was generally in nanoscale, varying from a few molecules to tens of nanometer.^[^ ^105^, ^114^ ^]^ Aqueous layer with such thickness range led to ice adhesion strength of ≈26 kPa, which was good but still beyond the requirement for practical anti‐icing application (lower than ≈10 kPa).^[^ ^14^, ^25^, ^26^ ^]^ To further increase the lubricant effects by interfacial aqueous lubricant layer, DAIS that could melt the interfacial ice and create thicker aqueous layer were developed. In a most straightforward manner, dynamic melting of ice contacting substrates could be initiated by chemicals (antifreeze liquid or salts) or thermal energy (magnetic thermal energy, electrothermal energy, and photothermal energy) that have long been used in active deicing techniques for pavement, aircraft, power line systems, and so on.^[^ ^4^, ^46^, ^121^, ^122^, ^123^, ^124^, ^125^, ^126^, ^127^, ^128^, ^129^, ^130^, ^131^, ^132^ ^]^ By contrast to the high costs and the detrimental environmental impacts of the traditional deicing methodologies, the recent approaches of introducing the active anti‐icing agents into passive anti‐icing substrates with dynamic change ice–substrate interfaces had shed new light on compromising solutions for ice removal with minimized energy/chemicals input.^[^ ^34^, ^45^, ^49^, ^53^, ^133^ ^]^

**Figure 9:**
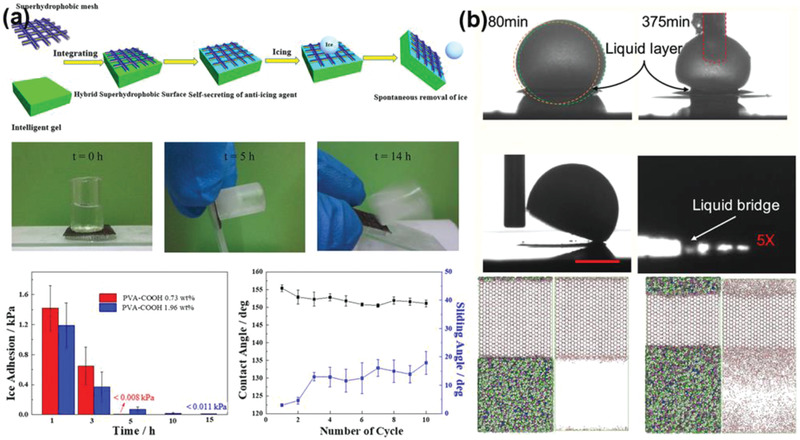
DAIS through dynamic interface melting. a) The hybrid superhydrophobic surfaces that dynamically secrete anti‐icing agents. Adapted with permission.^[^ ^45^ ^]^ Copyright 2019, American Chemical Society. b) The ionogel surfaces using ionic liquid for dynamic anti‐icing, and their atomistic models of ice melting. Adapted with permission.^[^ ^53^ ^]^ Copyright 2020, American Chemical Society.

It is well known that high ice adhesion strength is generally observed on superhydrophobic surfaces in humid environments, owing to strong interlocking between ice and the surface hierarchical structure of the surfaces.^[^ ^16^, ^17^, ^18^, ^28^, ^29^, ^30^, ^134^ ^]^ Using antifreeze agents on superhydrophobic surfaces to create liquid interface can provide a practical solution for weakening ice adhesion, as demonstrated by a newly fabricated superhydrophobic copper mesh with organogel that can dynamically secrete antifreeze agents.^[^ ^45^ ^]^ Specifically, PVA‐grafted succinic acid (PVA‐COOH) containing antifreezing agents (mixture of ethylene glycol and water) was applied on the copper mesh. The antifreezing agents were dynamically released at subzero temperature to the ice–substrate interface and melted the neighbouring ice. With the increasing thickness of the interfacial liquid layer, the ice adhesion strength on the surface deceased correspondingly with time. As shown in **Figure** **9**a, ice adhesion strength of PVA‐COOH (0.73 wt%) decreased from ≈1.3 kPa to <0.001 kPa with increasing holding time after ice formation from 1 to 5 h. For selected samples, the ice cube fell off automatically after 15 h on the substrate with small tilting angle, showing an excellent ice self‐removal capacity.

Ionic liquids were also selected as antifreeze agents for interface melting. Ionic liquids were commonly integrated in ionogels, and utilized in many fields ranging from solid electrolytes to drug release and to catalysis.^[^ ^135^, ^136^, ^137^, ^138^, ^139^, ^140^, ^141^ ^]^ Ionogel surfaces consisted of ionic liquid and polymer components were introduced for anti‐icing, as depicted in Figure 9b.^[^ ^53^ ^]^ In such ionogels, the polymer matrix endowed the substrate with hydrophobicity, and at the same time the embedded ionic liquid melted the contacting ice at the ice–substrate interface. If water droplet contacted the ionogel instead, the ionic liquid diffused from substrate to the interface and significantly depressed ice/frost formation. In either case, the ionic liquid resulted in an aqueous lubricant layer with high concentration ions at the interface, exhibiting significant anti‐icing performances. The lubricant layer on the one hand suppressed ice nucleation, on the other hand lowered the ice adhesion to the surfaces. The thickness of interfacial liquid layer on the ionogel surface increased with holding time, as the ice–substrate interface was dynamically melted as indicated in Figure 9b, which enabled easy detachment of frozen droplets. Interestingly, the liquid layer can have a macroscale thickness, as visual liquid bridges were observed between the detaching frozen droplets and the surfaces. The dynamic process of ice melting at the interface on ionogels was further revealed by atomistic modeling and molecular dynamics simulations, which provided a solid theoretical base for rational design of ionogel‐based anti‐icing surfaces. The dynamic interface melting through anti‐icing agent and ionic liquid can provide extremely low ice adhesion within several hours holding time, which however still need to be improved on their long‐term durability and timely ice removal performance.

**Figure 10:**
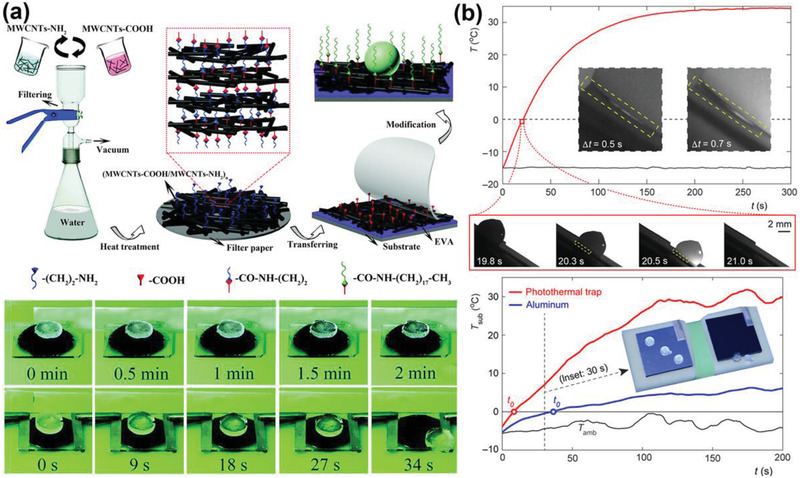
DAIS through dynamic interface melting. a) The superhydrophobic carbon nanotube surfaces with electrothermal effects. Real‐time anti‐icing testing experiments were showed in the figure. Adapted with permission.^[^ ^49^ ^]^ Copyright 2018, Royal Society of Chemistry. b) The photothermal trap utilizes solar illumination for ice mitigation. Adapted with permission.^[^ ^38^ ^]^ Copyright 2018, AAAS.

Utilizing thermal energy for interfacial ice melting is another accessible approach for creating nonfrozen water lubricating layer. The thermal energy can be generated from the substrate, and then transferred to melt the interfacial ice, as examples shown in **Figure** **10**a. Multiwalled carbon nanotubes (MWCNTs) with superior thermal‐conducting property were assembled into a layer‐by‐layer film through a vacuum‐assisted method. In the resulting superhydrophobic surfaces, excellent water repellency and special electrothermal effect for easy ice removal were observed.^[^ ^49^ ^]^ Specifically, the temperature of the substrate can be controlled by external voltage without hampering the surface superhydrophobicity. With an input voltage of 30 V, the ice–substrate interface was efficiently melted, leading to ice automatically sliding away in 34 s.

Using electrothermal surfaces for interfacial ice melting is effective, yet is still energy intensive. Nowadays, new surfaces with photothermal effects that harvest solar energy for interfacial ice melting have attracted special attentions.^[^ ^38^, ^132^, ^142^, ^143^, ^144^ ^]^ One eye‐catching example of these new surfaces, a so‐called photothermal trap, was fabricated recently. The photothermal trap surfaces consisted of a trilaminar structure, namely, a top solar radiation absorber layer for harvest illumination, a middle thermal spreader layer for lateral heat dispersal, and an insulator layer to minimize heat loss.^[^ ^38^ ^]^ Through this design, the surfaces can efficiently convert solar energy to heat in the substrate. As shown in Figure 10b, the frozen droplets started sliding away in 19.8 s with illumination on the substrate with tilting angle 30°. Remarkably, the substrate took a short time of 0.5 s to generate a thin liquid layer after the start of interfacial ice melting. In outdoor ambient anti‐icing tests, the substrate also exhibited highly encouraging anti‐icing performance, with frozen droplets started sliding away in 30 s in solar illumination intensity of ≈0.6 kW m^−1^. With longer solar illumination time and intensity, DAIS utilizing thermal energy for promoting ice removal not only melt the ice–substrate interfaces but also can melt the whole ice, which will be introduced in Section 4.4.

### 3.3 Dynamic Interface Generators

**Figure 11:**
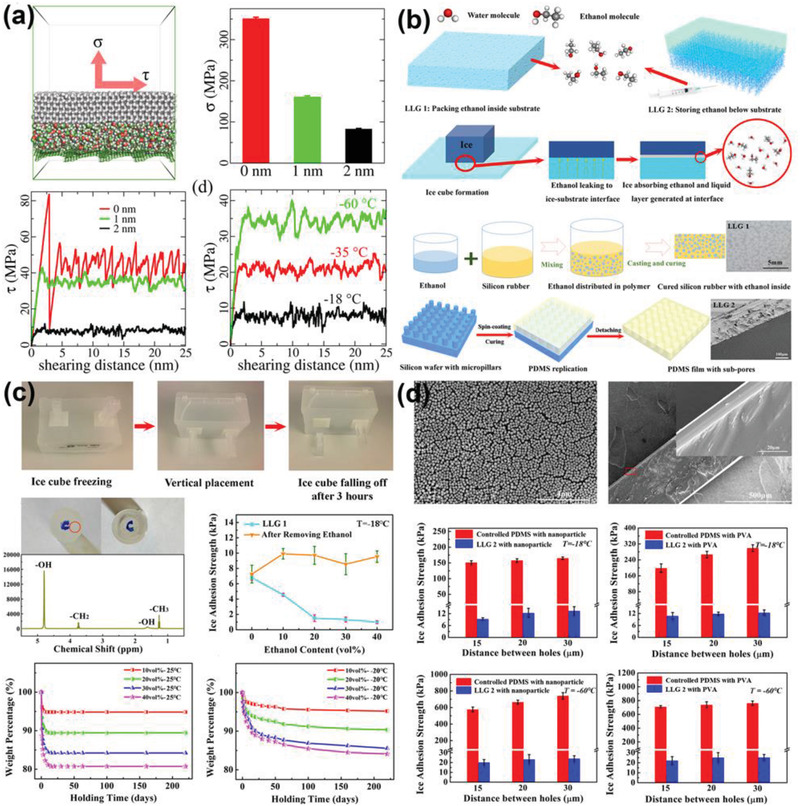
DAIS through dynamic interface generators. a) Interfacial ethanol layers of different thickness and their effects in reduction ice adhesion strength. b) The design principles and fabricated samples of the LLGs. c) The icephobicity and durability of LLG‐1. d) The icephobicity of LLG‐2 with various surfaces and under extremely low temperature. (a–d) Adapted with permission.^[^ ^35^ ^]^ Copyright 2019, Royal Society of Chemistry.

Durability, low ice adhesion, and extreme‐low temperature are three essential demands for practical applications of anti‐icing surfaces. It is still challenging for the current DAIS to meet all the three requirements. As discussed above, substrates that dynamically generate an interfacial aqueous lubricating layer can have improved durability thanks to lubricant regenerability.^[^ ^14^, ^25^, ^26^ ^]^ However, there is an exhaustion limit of these anti‐icing surfaces that the antifreeze agents can secrete. When the concentration gradient of chemicals disappeared, the antifreeze agent secretion stopped on these substrates, leading to poor durability in icing/deicing cycles. The anti‐icing surfaces with photothermal effects also lost their icephobicity at temperature lower than −50 °C. Subsequently, the interfacial aqueous layers tend to freeze at certain low temperature, which led to a sharp and dramatic increase in ice adhesion strength. For instance, the ice adhesion on substrates with interfacial aqueous layer can increase from ≈27 kPa to more than 400 kPa at temperature close to −60 °C.^[^ ^105^ ^]^ For anti‐icing at extremely low temperature, such as in the Arctic area, maintaining low ice adhesion on anti‐icing surfaces is a formidable task. A strategy of generating interfacial liquid layer at extremely low temperature and addressing anti‐icing in harsh environment was developed recently.^[^ ^35^ ^]^ Instead of generating pure aqueous layer for lubrication, ethanol was selected as the lubricant at the ice–substrate interface because of its low freezing point of −115 °C. The low freezing point of ethanol guarantees nonfrozen lubricating effects at extremely low temperature found in the biosphere. As verified via the atomistic modeling and molecular dynamic simulation shown in **Figure** **11**a, ethanol layer as thin as 2 nm at the ice–substrate interface can maintain low ice adhesion at −60 °C. In comparison, interfacial aqueous layer of the same thickness froze at a much high temperature, resulting in loss of lubrication effect. Based on the theoretical study, two liquid layer generators (LLGs) that can dynamically create ethanol layers at the ice–substrate interfaces were designed (Figure 11b). The first LLG was fabricated by packing ethanol into the substrates, termed LLG‐1, which can yield superlow ice adhesion of ≈1 kPa (samples containing 40 vol% ethanol). Because the ethanol layer was dynamically secreted from the substrate, the LLG‐1 had a continuous decrease in ice adhesion strength for spontaneous ice removing under its own weight. Specifically, ice adhered onto the LLG‐1 was detached by gravity in 3 h in the experiments. Most remarkably, the LLG‐1 was showed to have ethanol secretion lifetime of at least 250 days at −20 °C (Figure 11c). With more ethanol embedded in the substrate, the lifetime of ethanol secretion increased. For example, the LLG‐1 sample with 40 vol% ethanol can have a long functioning lifespan of 593 days. In order to further extend the ethanol exhaustion time, a second LLG (LLG‐2) with subporous layers was designed for the possibility of replenishing ethanol in the substrates, which showed the same outperforming anti‐icing properties. The LLG‐2 strategy was applied on various surfaces, including the contaminated ones with particles and other hydrophilic components. Surprisingly, the contaminated LLG‐2 surfaces had superlow ice adhesion strength of ≈10 kPa, which was expected to further decrease with increasing secreted ethanol layer. The most attractive properties of the LLGs were their unprecedented low ice adhesion strength at extremely low temperature (Figure 11d). By introducing the ethanol lubricating layer at interfaces, the ice adhesion strength on the same surfaces decreased from 709.2–760.9 to 22.1–25.2 kPa at −60 °C, which verified the LLGs as a viable candidate for anti‐icing applications at harsh temperature. Thus, the LLGs are the first dynamic substrates that have the potential to meet the above‐mentioned three anti‐icing requirements of realistic applications. It should be note that the lifespan of LLG using ethanol as lubricant greatly depends on the environmental temperature. High temperature will intensify ethanol evaporation and challenge the function of LLG.

### 3.4 Interfacial Crack Initiators

**Figure 12:**
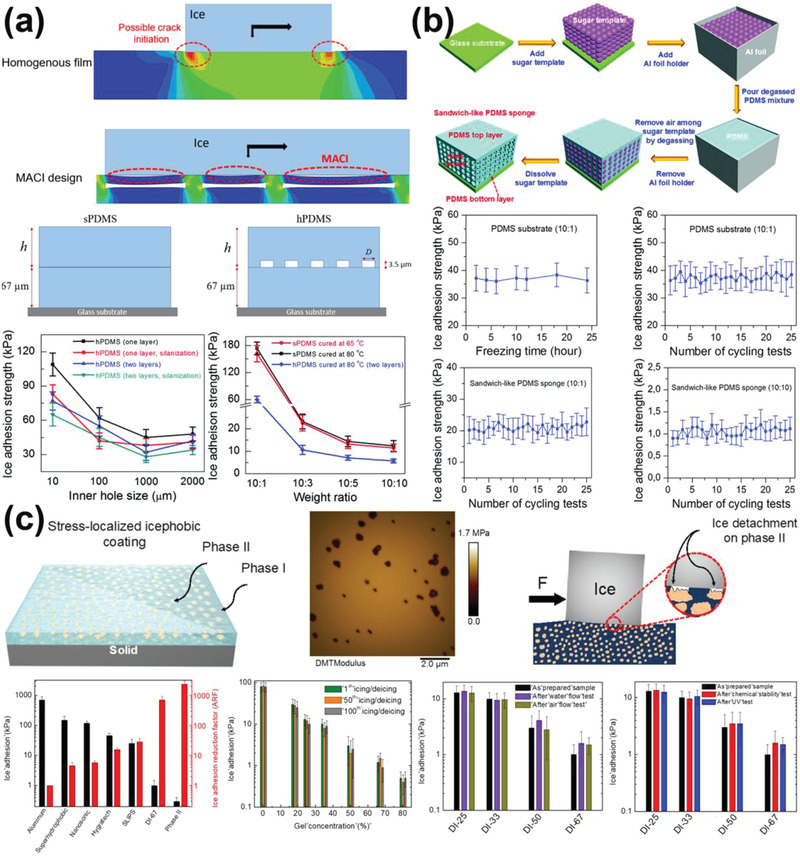
DAIS through interfacial crack initiators. a) The mechanism of multiscale crack initiator (MACI) for superlow ice adhesion and their experimental results. Smooth films without structures and smooth films with internal holes were named as sPDMS and hPDMS, respectively. Adapted with permission.^[^ ^14^ ^]^ Copyright 2017, Royal Society of Chemistry. b) The fabrication of sandwich‐like PDMS sponges with MACI and the resulting anti‐icing performances. Adapted with permission.^[^ ^149^ ^]^ Copyright 2018, Royal Society of Chemistry. c) The stress‐localized surfaces and their ice adhesion testing results. Adapted with permission.^[^ ^40^ ^]^ Copyright 2019, Royal Society of Chemistry.

Dynamic interface change can also be a result of interfacial cracking due to stress concentration. According to classic fracture mechanics theory, ice adhesion strength (*τ*_c_) is governed by $\tau_{c}=\sqrt{E^{*}G/\pi a\Lambda }\;$, where *G* is the surface energy, E* is the apparent bulk Young's modulus, *a* is the total length of crack, and Λ is a nondimensional constant. Therefore, generating cracks at the ice–substrate interface is a promising approach for low ice adhesion. Following the fracture mechanics principle, surfaces containing crack initiators at the ice–substrate interfaces were fabricated, which can enhance crack generation and efficiently reduce ice adhesion.^[^ ^14^ ^]^ In order to promote the generation of cracks at the ice–substrate interface, three crack initiators on different length scales were first identified, namely, nanoscale crack initiator (NACI) by surface atomistic and chemistry characteristic, microscale crack initiator (MICI) by the hierarchical surface structures and macroscale crack initiator (MACI) by interface stiffness inhomogeneity.^[^ ^14^ ^]^ NACI underlies negative and weak affinity of the surface to ice and aids debonding at the ice–substrate interface, which is widely observed in hydrophobic surfaces used for anti‐icing.^[^ ^101^, ^145^, ^146^ ^]^ MICI can be taken as the microvoids under the so‐called “Cassie” ice on superhydrophobic surfaces and serve as microcracks for ice detachment from the surfaces.^[^ ^28^, ^147^, ^148^ ^]^ Both NACI and MICI are believed to have their limitations for achieving superlow ice adhesion (defined as ice adhesion strength lower than 10 kPa).^[^ ^14^ ^]^ By contrast, MACI is the only crack initiator that can maximize macroscale crack size at the ice–substrate interfaces and predominantly facilitate ice removal.^[^ ^14^ ^]^ As shown by finite element‐based simulation in **Figure** **12**a, porous subsurface structures for MACI consist of significantly larger number of crack initiation sites along the ice–substrate interface than the cases with a homogenous substrate. Correspondingly, PDMS coatings with MACI showed superlow ice adhesion of 5.7 kPa, much lower than their counterparts, as the coating sketches and results showed in Figure 12a.

The novel MACI mechanism enabled by subsurface structures thus provided a new route for design icephobic surfaces. One low‐cost yet effective fabrication strategy of realizing MACI was using sponge structure directly.^[^ ^149^ ^]^ As shown in Figure 12b, sugar was used as a sacrificial template for preparing sandwich‐like PDMS‐based sponge substrates. Because of the high porosity, the MACI effect of the sponge‐type substrates was greatly enhanced. Furthermore, the elastic modulus of the sponge substrates was intrinsically low, which also beneficially contributed to the overall surface icephobicity. The PDMS sponges showed a remarkable superlow ice adhesion strength of ≈0.8 kPa and stable long‐term ice adhesion strength of ≈1 kPa after 25 icing/deicing cycles.

One can see that stiffness inhomogeneity in the substrate is the key to the success of MACI. Other anti‐icing substrates utilizing surface stiffness inhomogeneity to achieve low ice adhesion can be considered to rely on the same mechanism of MACI. For example, stress‐localized surfaces for lowing ice adhesion were developed by surface inhomogeneity programming.^[^ ^40^ ^]^ Specifically, the stress‐localized surface contained two phases, phase I and phase II, as shown in Figure 12c. The phase I was polymer with relatively high elastic modulus, while the phase II was stress‐localized materials with low elastic modulus. In such a setup, the phase II served as crack initiator, similar to pores in the PDMS‐based sponge with MACI, for ice detachment under mechanical loading, which rendered the stress‐localized surface one of the lowest ice adhesion strength surfaces reported so far (in the order of 1 kPa). It is worth noting that the stress‐localized surface had excellent mechanical durability thanks to the high elastic modulus of phase I. The surfaces with 80 vol% phase II concentration showed a stable long‐term ice adhesion strength lower than 1 kPa after 100 icing/deicing test. The icephobic coatings that exposed to high speed flow of water and air for one month showed no change in ice adhesion, which made it a good candidate for outdoor surfaces in practical conditions. Further durability of this surface was examined by abrading surface with sandpaper and filing, immersing surface into chemical solutions with pH in range of 1–13, and exposing surface to UV radiation for 500 h. No change in the integrity, property or ice adhesion was observed. Therefore, the stress‐localized surfaces provide alternative choices for anti‐icing surfaces that are durable to mechanical, chemical, and environmental challenges. Utilizing stiffness inhomogeneity to dynamically introduce interfacial cracks for easy ice removal can act as one of the most promising method for fabricating durable icephobic coatings. However, large scale fabrication methods that can promote the surfaces from laboratory scale to real application are still missing.

## 4 Dynamic Ice

### 4.1 Ice Growth Inhibitors

The properties of ice on a surface are dynamically changing with time and the environmental conditions, which can also be utilized as an anti‐icing strategy. In outdoor environment, ice nucleation is generally inevitable due to low temperature, long icing time, and surface contaminations. Once ice started to form on exposed surfaces, ice growth/propagation control was believed to be more important to tackle the ice accumulation problem and thus one important focus of anti‐icing surface design.^[^ ^150^, ^151^, ^152^ ^]^ Natural organisms like insects, fishes, and plants utilizing antifreeze proteins (AFPs) to survive subzero temperature are the vivid examples.^[^ ^40^, ^153^, ^154^ ^]^ AFPs can not only suppress freezing‐point but also inhibit ice growth and recrystallization,^[^ ^155^, ^156^, ^157^, ^158^, ^159^ ^]^ thanks to their Janus properties. All the AFPs have ice‐binding faces (IBFs) and nonice‐binding faces (NIBFs).^[^ ^160^ ^]^ In solution, AFPs preferentially bind to any ice crystals with IBFs and leave the NIBF to face liquid water, which results in microcurvatures of ice–water interfaces. Due to the so‐called Kevin effect, such curved ice–water interfaces inhibit ice growth.^[^ ^161^ ^]^ Inspired by the discovery that ice growth was regulated by the AFPs absorption onto the basal/prism planes, novel materials featuring the same property of preferential binding to ice surfaces were discovered, including graphene oxide (GO), oxidized quasi‐carbon nitride quantum dots, safranine molecules, and so on.^[^ ^51^, ^162^, ^163^, ^164^, ^165^, ^166^, ^167^, ^168^ ^]^ Although the inhibition of ice growth in solutions containing these new materials has been widely reported, applying the same strategy on the design of anti‐icing surfaces is at the early stage of development. AFPs were directly immobilized onto aluminum, and led to delayed frost/ice formation for at least 3 h, showing significant anti‐icing potential.^[^ ^169^ ^]^ The binding faces of AFPs, IBFs, and NIBFs were screened for freezing‐point depression, indicating only the NIBFs decreased freezing temperature.^[^ ^48^ ^]^ Despite that the related studies confirmed the potential of AFPs in ant‐icing applications, more in‐depth investigations are still needed for elucidating their effects on the overall surface icephobicity performance.

**Figure 13:**
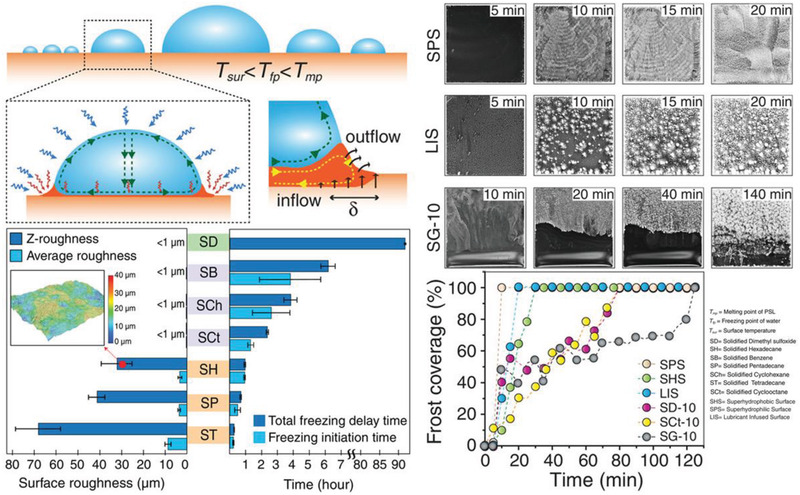
DAIS through ice growth inhibitors. Phase‐switching liquids used for inhibiting ice growth, and their freezing delaying time. Adapted with permission.^[^ ^170^ ^]^ Copyright 2019, Wiley‐VCH.

Phase‐switching liquids (PSLs) containing cyclohexane (SCh) were used for inhibiting ice growth, for their property of holding ice melting temperature (*T*_mp_) above water freezing point (*T*_fp_).^[^ ^170^ ^]^ When vapor condensed on a solidified PSL surface, the latent heat released during the condensation was trapped in the droplets. The temperature increased at the solidified cyclohexane (SCh) and air interfaces could attain ≈5 °C (**Figure** **13**). The increased temperature thus led to melting of PSL in contacted region. By comparing the freezing initiation time and total freezing delaying time, PSL‐coated surfaces performed excellently in inhibiting ice growth.^[^ ^170^ ^]^ The best sample showed sustained ice growth delay for more than 96 h, 300 times longer than the superhydrophobic surfaces. The PSLs can also be infused into porous surfaces and form SLIPS. The infused samples with 10 µm spacing microstructured (SG‐10) surfaces dynamically inhibited frost propagation for more than 140 min, around seven times longer than LIS. It should be noted that the reported PSL chemicals, such as benzene, were highly toxic. Other safe and environmentally friendly phase‐switching liquids that have melting temperature close to the water freezing point are desired.^[^ ^36^ ^]^

**Figure 14:**
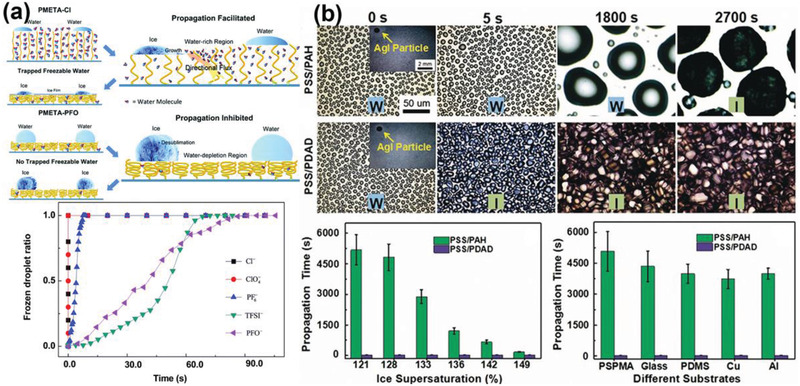
DAIS through ice growth inhibitors. a) Ion‐specific ice propagation on polyelectrolyte brush surfaces. Adapted with permission.^[^ ^171^ ^]^ Copyright 2017, Royal Society of Chemistry. b) Ice propagation on polyelectrolyte multilayer surfaces, “W” and “I” are denoted as “water” and “ice,” respectively. Adapted with permission.^[^ ^52^ ^]^ Copyright 2017, Wiley‐VCH.

Counterions at the ice–substrate interface can also inhibit ice growth by dynamic melting.^[^ ^39^, ^171^, ^172^, ^173^ ^]^ Poly([2‐(methacryloyloxy)ethyl]trimethylammonium chloride)‐Cl (PMETA‐Cl) brush was synthesized on silicon wafers, which can accommodate different ions via ion exchange.^[^ ^171^ ^]^ Brushes with counterions of Cl^−^, ClO_4_^−^, PF_6_^−^, TFSI^−^, and PFO^−^ were investigated, showing hydrophobicity Cl^−^ < ClO_4_^−^ < PF_6_^−^ < TFSI^−^ < PFO^−^. It was found in the study that ice propagation was efficiently on the highly hydrated PMETA‐Cl and PMETA‐ClO_4_ brushes, while much longer time were needed for ice form on brushes with counterions of PF_6_^−^, TFSI^−^, and PFO^−^. Thus, it was assumed that water molecules in the hydrophilic polymer brush (PMETA‐Cl and PMETA‐ClO_4_) moved toward the ice growth fronts when freezing occurred and consequently promoted ice propagation. By contrast, brushes with PF_6_^−^, TFSI^−^, and PFO^−^ counterions and higher hydrophobicity could result in “water depletion regions,” which reduced the freezable water molecules and suppressed ice propagation (**Figure** **14**a).

To understand the mechanism that control the ice propagation, ice propagation behaviors on polyelectrolyte multilayer (PEM)‐coated surfaces were investigated.^[^ ^52^ ^]^ The PEMs were ideal for this purpose, because the outmost layers determined the surface properties and water concentrated only in the outside layer. Two types of PEMS, the poly(sodium 4‐styrenesulfonate)/poly(allylamine hydrochloride) (PSS/PAH) and the PSS/poly(diallyldimethylammonium chloride) (PSS/PDAD) coatings were fabricated by tuning the compositions of polyelectrolyte pairs during the layer‐by‐layer deposition. The PDAD had polyelectrolyte interacting more strongly with water in the outmost layer than that in the PAH. Consequently, ice propagation time on the PSS/PAH was two orders of magnitude longer than on the PSS/PDAD with same number of deposition layers. The ice propagation time can be tuned by simply change the composition of the outmost layers, but not by the number of deposition layers. Counterions and their concentration also showed positive effects on ice inhibiting performance on the PEMs. The results indicated that ice propagation was inhibited by depressing the water molecules trapped in the outmost layer of polyelectrolyte coatings. More importantly, the ice growth inhibition properties of PEMs layer in real environment was also investigated in the related studies (Figure 14b).^[^ ^52^ ^]^ With silver iodide aqueous droplets (AgI particle, 0.1 µL) placed on the PEMs acting as the artificial ice nuclei, the PSS/PAH samples were still able to delay ice propagated for 2700 s, demonstrating the robustness and wide applicability of their anti‐icing performances. Suppressing ice propagation through counterions is an effective way to avoid rapid frost coverage on whole surfaces comparing to other strategies. However, the ice propagation delaying time still need to be extended for practical usage.

### 4.2 Ice Growth Controlling

**Figure 15:**
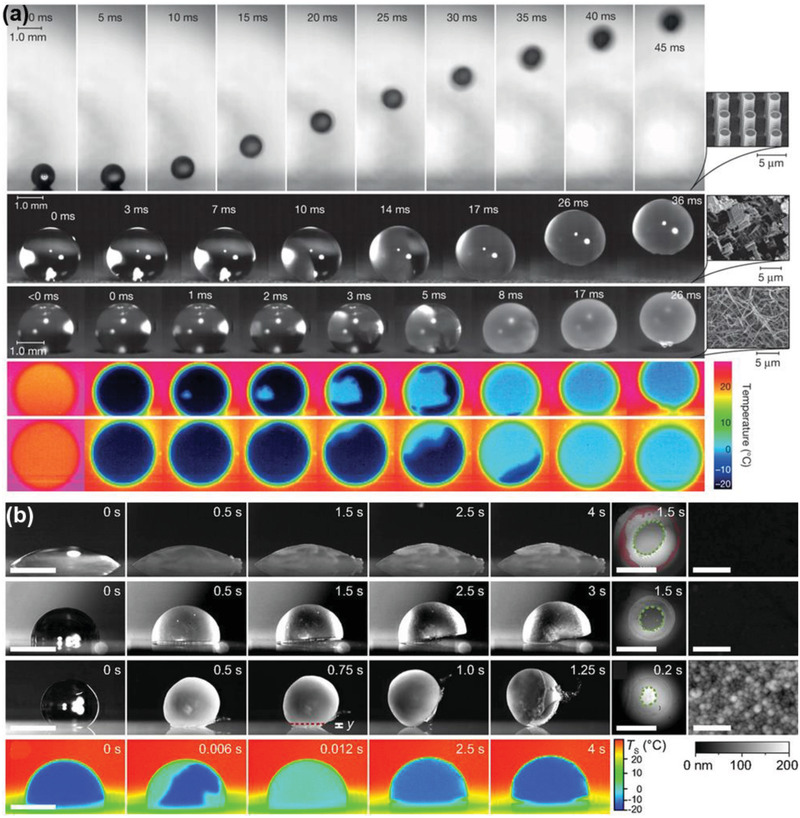
DAIS through ice growth controlling. a) Spontaneous droplet levitation in freezing from a wide range of surfaces under low‐pressure and low‐humidity conditions. Adapted with permission.^[^ ^174^ ^]^ Copyright 2015, Nature Publishing Group. b) Self‐dislodging behavior of frozen droplets on hydrophilic, hydrophobic, and superhydrophobic surfaces. Adapted with permission.^[^ ^54^ ^]^ Copyright 2017, National Academy of Sciences of the United States of America.

Controlling ice growth in a defined pattern on a surface can also be an effective approach to achieve icephobicity, given that ice formation is unavoidable after growth inhibition for a limited period. It was found that water droplets on superhydrophobic surfaces showed spontaneous levitation and trampoline‐like bouncing behavior in a low‐pressure environment.^[^ ^174^ ^]^ Such phenomenon was caused by the overpressure beneath the droplet originated from fast vaporization and the countering surface adhesion restricting the vapor flow. Because strong vaporization led to high degree of cooling on a supercooled droplet, a rapid recalescence freezing was initiated at the free surface.^[^ ^152^, ^175^ ^]^ The latent heat released during the freezing increased the droplet temperature to the equilibrium freezing temperature (0 °C). Under the low‐pressure and low‐humidity conditions, a sudden increase in the vaporization of droplets surfaces resulted in rapid increasing in the overpressure beneath the droplet, which enabled self‐levitation of the droplet when freezing happened. Such phenomenon can be observed on various surfaces with different textures, as shown in **Figure** **15**a, which suggests the potential of controlling ice growth for spontaneous ice removal.

The freezing‐driven ice‐removal mechanism was further explored on various surfaces.^[^ ^54^ ^]^ It was found that the key for self‐dislodging of frozen droplets lied in the freezing orientating process. Specifically, the successful self‐dislodging required free spherical surface of the droplet to be solidified first, forming an ice shell on the external surfaces while maintaining the droplet–substrate contacted region remained liquid. As the subsequent freezing phase boundary moved inward in the droplet asymmetrically, the volume of droplet expanded during the solidification process and caused mass displacement toward the unsolidified droplet–substrate interface. On nonwetting surfaces with pinned ice–liquid–vapor contact line, the displaced mass had no extra space to spread in the final state of solidification and lifted the droplet upward. This dislodging process was observed on various substrates with a wide range of wettability (from hydrophilic to superhydrophobic) and topography, as shown in Figure 15b. Such self‐dislodging behavior of frozen droplets provided a universal concept for design ice free surfaces through controlling asymmetric freezing dynamics. Although the above phenomenon was observed under low pressure which might limit its application scope, the self‐dislodging brought insight into the freezing fundaments and inspired the further development of dynamic icephobic surfaces through controlling ice growth behaviors.

**Figure 16:**
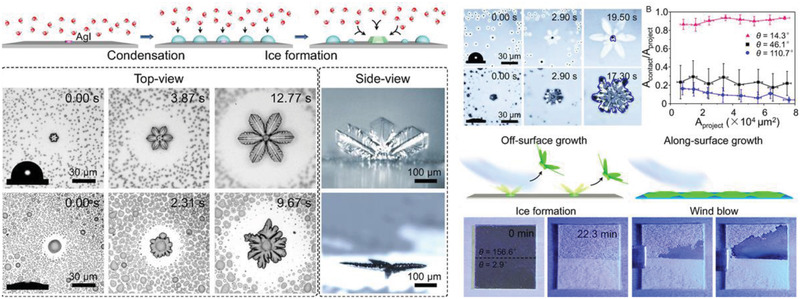
DAIS through ice growth controlling. Ice growth pattern on surfaces with different wettability and their anti‐icing performance. Adapted with permission.^[^ ^13^ ^]^ Copyright 2017, National Academy of Sciences of the United States of America.

By controlling the multicrystal ice growth pattern on exposed surfaces, another important design for novel dynamic ice repellency was also developed recently.^[^ ^13^ ^]^ By depositing silver iodide (AgI) nanoparticles as ice nucleation active sites on surfaces, ice nucleation was controlled to occur concurrently. It was found that the ice crystals on a hydrophilic surface (with water contact angle of 14.5°) possessed an along‐surface growth (ASG) mode. However, the ice on the hydrophobic surface (with water contact angle of 107.3°) exhibited an off‐surface growth (OSG) mode (**Figure** **16**). On smooth surfaces, there was an ASG‐to‐OSG transition of ice growth mode at surface water contact angle of *θ* = 32.5° ± 1.9°. The ASG‐to‐OSG transition was also observed on porous surfaces at different water contact angle, suggesting the ubiquity of this behavior on various structured surfaces. The growth mode of ice crystals significantly affected the contact area (*A*_contact_) between ice and its substrate, and subsequently the ice adhesion strength. In the OSG mode, the optimal *A*_contact_ was only around 10% of projected area (*A*_project_). By contrast, the ASG mode resulted in almost full ice contact of the surface project area (*A*_contact_/*A*_project_ ≈ 90%). In ice‐removal tests, the OSG ice was easily blown away by a wind breeze with velocity of 5.78 m s^−1^ at −3 °C, while the ASG ice remained firmly on the surface. This unique off‐surface growth mode provides alternative ways for assisting surface frost removal, but further investigation is needed for testing on the icephobicity of the surface against larger water drops.

### 4.3 Ice‐Free Zone Programming

**Figure 17:**
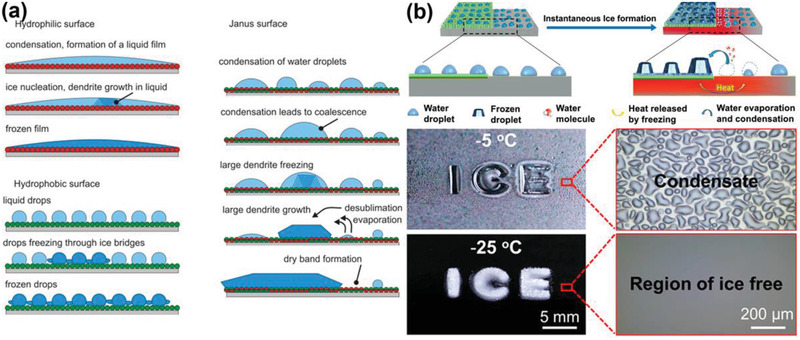
DAIS through ice‐free zone programming. a) Ice formation mechanism on rough hydrophilic, hydrophobic, and Janus surfaces. Adapted with permission.^[^ ^176^ ^]^ Copyright 2016, American Chemical Society. b) Patterned polyelectrolyte coatings for dynamic controlling large scale ice‐free regions. Adapted with permission.^[^ ^177^ ^]^ Copyright 2020, American Chemical Society.

Because the dynamic ice growth patterns are important for anti‐icing, recent investigations further strived to program ice growth on the whole surfaces through surface chemistry/structure deign.^[^ ^50^, ^176^, ^177^, ^178^ ^]^ On surfaces fabricated by assembling poly(poly(ethylene glycol) methyl ether methacrylate)/PDMS (P(PEGMA)/(PDMS)) Janus particles, ice was found to grow into ice crystals/dendrites in certain regions, leaving the other regions dry and clean.^[^ ^176^ ^]^ The underlying mechanism of the distinct ice growth pattern on the Janus surfaces was elucidated, as depicted in **Figure** **17**a. For the hydrophilic regions of the Janus surfaces, water condensed into liquid film and formed continuous frozen films. While on the hydrophobic regions, water droplets formed in condensation, which resulted in frozen droplets and ice bridges. The icing process on the Janus surfaces followed a unique process. First, condensed droplets froze inhomogeneously on the surface. Specifically, ice crystals emerged likely in the large liquid droplets on the hydrophilic regions of the surface. The frozen droplets then affected the surrounding regions. Instead of freezing, the small droplets on the hydrophobic clustered around the frozen droplets and disappeared through evaporation. Then, desublimation of vapor to ice assisted the further growth of the already existing frozen droplets into dendrites, which ultimately led to a dry band around the large frozen dendrites, namely, ice‐free zones. Because of the ice‐free zones and small ice contact area, the Janus surfaces exhibited low ice adhesion strength of 56 kPa.

The guided ice growth pattern on Janus surfaces suggested that surface chemistry design could dynamically create ice‐free zone. However, the locations of ice‐free zones on a surface were mostly random. In a recent report, ordered and large scale ice‐free zones taking up ≈96% of the entire surface area were programmed and created on surfaces with patterned polyelectrolyte.^[^ ^177^ ^]^ On these patterned surfaces, icing was designed to initiate from the polyelectrolyte domains and propagated atop, as shown in Figure 17b. The condensation of vapor into water droplets on the polyelectrolyte released latent heat, which led to significant temperature increase atop the substrate close to the frozen droplets. This freezing‐driven thermal effect resulted in further water evaporation around the frozen droplets. Because the saturated vapor pressure of water droplets was higher than ice, the newly formed ice was guided to grow exclusively on the patterned polyelectrolyte domains through desublimation.^[^ ^52^, ^109^, ^160^, ^171^ ^]^ By contrast, ice growth at the ice‐free zone was inhibited. Thus, ice accumulated on the frozen area was accompanied by the consumption of condensed droplets and resulted in the continuous expansion of the ice‐free zones. It was showed in the experiments that the fraction of ice‐free region could be tuned by the grafting density and pattern of polyelectrolyte brushes. By using polyelectrolyte multilayer and polyelectrolyte hydrogel for patterning surfaces, ice‐free regions occupying ≈96% and ≈88% of the total surface area were achieved, respectively.

**Figure 18:**
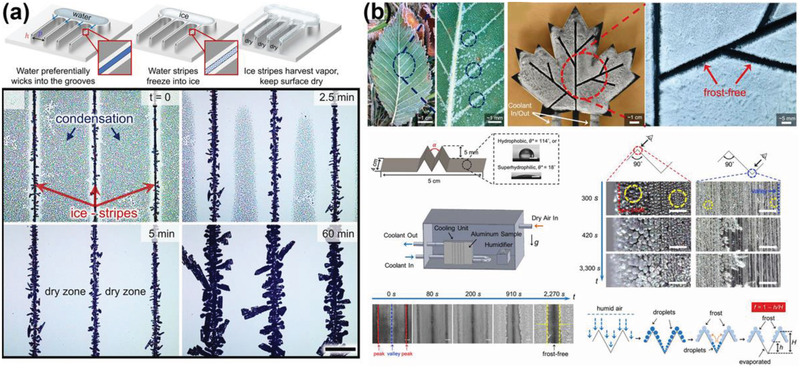
DAIS through ice‐free zone programming. a) DAIS using microscopic ice patterns. Adapted with permission.^[^ ^178^ ^]^ Copyright 2018, American Chemical Society. b) Microtextured surfaces with capacity of dynamic forming ice‐free zones. Adapted with permission.^[^ ^50^ ^]^ Copyright 2020, National Academy of Sciences of the United States of America.

Besides patterning in surface chemistry, microstructures can also be utilized for programming ice‐free zone on surfaces. As shown in **Figure** **18**a, precisely designed micropatterned surfaces were used for programing ice growth aim of using ice to fight ice.^[^ ^178^ ^]^ The unique feature of such surfaces was the introduction of water into microstripes on the surface. Under cooling, the water stripes on the surface froze first, serving as frozen regions. The formation of ice‐free and dry area on the surface followed the same process as discussed above, which led to as high as 90% of the total surface area as dry‐zone (or ice‐free zone). Interestingly, it was found that the growth rate of frost on the ice‐stripes was one order of magnitude lower than that on smooth solids. In short, the prefrozen ice pattern not only protected the other region from icing but also restrained ice propagation.

Natural surfaces can also program ice formation in winter, namely, ice accumulation on convex veins is more preferred than flat zones. Inspired by such phenomena, artificial leaves with ice‐free regions were designed recently.^[^ ^50^ ^]^ The microtextures on the artificial leaves resulted in large scale ice‐free area without predesigned water/ice stripes, affecting both water condensation and ice formation on the surface. As water molecules in a supersaturated ambient air diffused more easily to the peaks than to the valleys, the artificial leaves accumulated more and larger droplets on top of its microtexture, where the freezing of droplets initiated at lower temperature. After ice formation on the peaks of the microtextures, the low vapor pressure led to the evaporation of droplets and created stable ice free zones in the valleys, as shown in Figure 18b. Combining the experiments and simulations, an important structure parameter *α*, namely, the vertex angle, was proposed to quantitatively correlate to the percentage of ice‐free regions and the microtexture on the surface. The relationship between *α* and frost coverage was mapped under various ambient humidity, providing significant instructions for constructing ice‐free regions through surface design and structure fabrication. These surfaces with programmed ice‐free zone can be used to protect specific area from icing. Nevertheless, challenges like maintaining the function after surface contaminations and enabling large area of surfaces free from ice in practical application still need to be addressed.

### 4.4 Dynamic Ice Melting

Ice melting on anti‐icing surfaces is another important aspect of dynamic ice change for avoiding unwanted ice accumulation.^[^ ^179^, ^180^, ^181^ ^]^ Surfaces that can dynamically melt ice were commonly created by integrating materials with thermal effects, including electrothermal, near‐infrared photothermal, magnetothermal, and solar photothermal effects.^[^ ^133^, ^144^, ^179^, ^180^ ^]^

**Figure 19:**
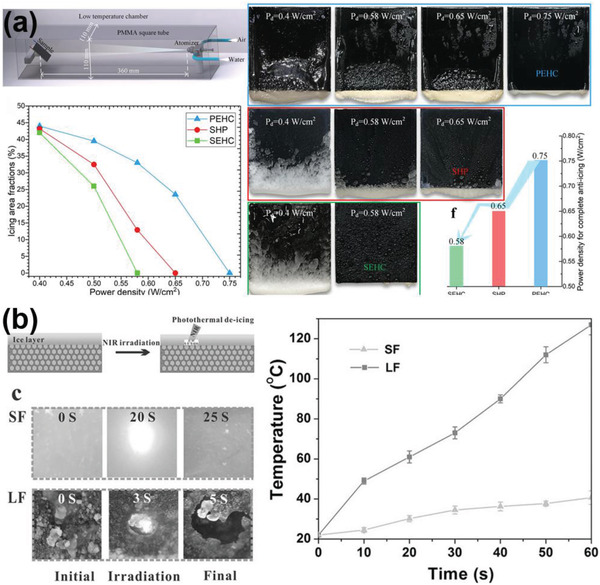
DAIS through dynamic ice melting. a) Dynamic anti‐icing performance of SLIPS combined with electrothermal effect. Adapted with permission.^[^ ^133^ ^]^ Copyright 2019, Elsevier Publishing Group. b) Integrating self‐lubrication and near‐infrared photothermal generation for dynamic ice melting. Adapted with permission.^[^ ^179^ ^]^ Copyright 2015, Wiley‐VCH.

The use of electrothermal effect for tackling icing problems has a long history. In the last decades, numerous active deicing surfaces with additive electrothermal effects were developed, which consumed electrical energy for dynamic ice melting.^[^ ^182^, ^183^, ^184^, ^185^, ^186^, ^187^ ^]^ Passive icephobic surfaces, such as superhydrophobic and the SLIPS surfaces, can also integrate active thermal effects for anti‐icing, as introduced in Section 3.2. By enhancing the power density on such surface, ice can be dynamically and constantly melted at the ice–substrate interface until being fully consumed. Recently, a so‐called slippery liquid infused porous electric heating coating (SEHC) was prepared, and contained MWCNTs as electrothermal generators.^[^ ^133^ ^]^ The SEHC also contained lubricating oil with high thermal conductivity that enabled reduction of ice adhesion strength from 1940 to ≈58 kPa. The obvious advantage of using SEHC instead of superhydrophobic surface was the high thermal conductivity of the infused lubricant, which was nearly one order of magnitude higher than that of air, and thus also enabled higher electrothermal efficiency. With power density of 0.58 W cm^−2^, ice on SEHC surface was melted completely while ice on superhydrophobic surface was remained (**Figure** **19**a). In another study, the lubricant infused surfaces were integrated with near‐infrared photothermal effect for dynamic melting ice.^[^ ^179^ ^]^ The lubricant infused film on the surfaces showed much lower ice adhesion strength of ≈25 kPa than that of surfaces with smooth film (≈506 kPa). Surfaces with embedded Fe_3_O_4_ nanoparticles (0.5 wt%) into the substrate was prepared for melting surface ice. The temperature of such surfaces increased for more than 50 °C in 10 s under irradiation, owing to the excellent near‐infrared thermal response of the Fe_3_O_4_ nanoparticles. In an ice‐melting test, the ice layer on the surface can be melted with ambient temperature below −5 °C (Figure 19b).

**Figure 20:**
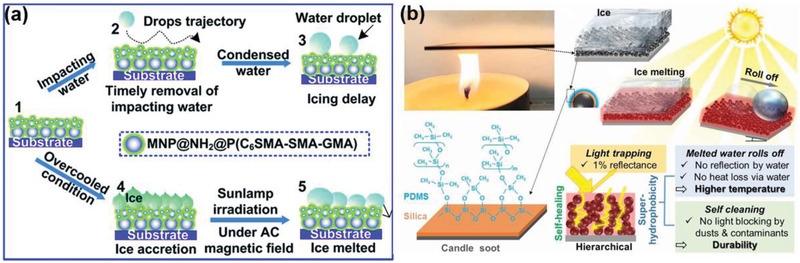
DAIS through dynamic ice melting. a) Magnetic particles based superhydrophobic surface for ice melting. Adapted with permission.^[^ ^180^ ^]^ Copyright 2015, Royal Society of Chemistry. b) Superhydrophobic photothermal icephobic surfaces based on candle soot. Adapted with permission.^[^ ^144^ ^]^ Copyright 2020, National Academy of Sciences of the United States of America.

The Fe_3_O_4_ nanoparticles were not only a good medium for photothermal generation, but also possessed excellent magnetothermal effects. Superhydrophobic coatings containing Fe_3_O_4_ nanoparticles can melt ice under magnetic field and sunlamp irradiation (**Figure** **20**a).^[^ ^180^ ^]^ Under a magnetic field of 7.8 kW, the hybrid surface containing 50 wt% Fe_3_O_4_ nanoparticles showed an increase in temperature of ≈21 °C in 25 s. At the meantime, the same surface under sunlamp irradiation (75 W) increased 13 °C in 5 min.

The dynamic ice melting surfaces described above by using electric power, near‐infrared irradiation, and magnetic field were attractive. However, these approaches were partially costly. The photothermal trap surfaces that used solar irradiation energy for ice melting were outstanding candidates and have raised great interests.^[^ ^38^, ^132^, ^142^, ^143^, ^144^, ^188^, ^189^ ^]^ Despite the complex design, these surfaces are efficient in ice melting. For instance, superhydrophobic icephobic surfaces with photothermal effects by using cheap candle soot was newly fabricated.^[^ ^144^ ^]^ Because of the hierarchical structure and the black color, the candle soot has excellent photothermal conversion efficiency.^[^ ^190^, ^191^, ^192^, ^193^, ^194^, ^195^, ^196^ ^]^ As shown in Figure 20b, the candle soot coated surfaces possessed a synergy of robust superhydrophobicity and highly efficient light trapping, which enabled surface temperature increase of ≈53 °C under 1 kW m^−2^. In a frost‐melting test at −30 °C, the accumulated frost started melting after illuminating 60 s and the surface became free of ice in 720 s, demonstrating superior dynamic ice melting performances. Despite the efficient interface melting even ice melting performance, challenges are remained for the electrothermal and photothermal enhanced surfaces. First, huge thermal input is required to remove vast ice on surfaces. Second, light irradiation is difficult to reach the surface for initiating photothermal effects with heavy snow/ice coverage. Therefore, efforts that can enhance the efficiency of electrothermal/photothermal effects in harsh conditions need to be made.

## 5 Conclusions and Perspectives

Unwanted icing is one of the long‐lasting challenges accompanying mankind in the whole civilization history. It is unfortunate that the most common ways utilized today to combat unwanted icing are still the traditional deicing methodologies involving direct mechanical forces, energy‐intensive thermal treatments or costly chemical handling. Despite that the passive anti‐icing concept has been proposed for more than a decade, automatic ice removal on application scale or at any industrial technology readiness level is still unavailable. Nevertheless, there are significant successes in the anti‐icing materials related researches, especially on the superlow ice adhesion surfaces that enable natural forces for effective ice removal. Although challenges such as durability, anticontamination, functioning at extremely low temperature and harsh conditions need to be addressed, the upscale of such anti‐icing surfaces from laboratory does promise a bright future. In concluding this review, we summarize the dynamic design principles of anti‐icing surfaces through enabling dynamic changes in the chemical/physical states of the ice/substrate/ice–substrate interface with tailored functions by the three DAIS categories, namely, surfaces with dynamic substrates, dynamic interfaces, and dynamic ice.

Owing to the abilities of response to stimuli, DAIS with dynamic substrates possess highly appealing potentials. This type of anti‐icing surfaces can utilize various stimuli like temperature, magnetic field, and light for enabling surfaces icephobicity, although not all external stimuli are available in harsh icing situations. For making more practical DAIS in this category, taking the advantages of the intrinsic properties of water and ice for triggering responses of the substrates is the most feasible approach, given that water and ice are always the existing component in the anti‐icing operations. It can be envisioned that novel DIAS with substrates responding sensitively to the interfacial water/ice in cold temperature can provide sensible possibilities in application.

DAIS with dynamic interfaces mainly focus on the ice–substrates interaction. To enable dynamic evolution of the interface, the following two pathways are recommended. The first is to enable dynamic change at the interface states, either by generating new interlayers to replace the ice–substrate interfaces or by melting the interface from rigid solid to lubricating liquid. The second is to enable dynamic evolution of the interactions between ice and substrates. This will require better understanding on the fundamental interactions between ice and solid. New interface layers and lubricating layers no doubt have reduced ice–substrates interactions. However, the practical approach of utilizing the intrinsic properties of ice and solids for lowering ice–substrates interactions is still missing. Further investigations that can accelerate such evolutions and apply them for effectively weakening ice adhesion are required.

The current DAIS with dynamic ice indicates that ice evolution after formation is not independent from surface properties. Both structures and chemical components of the surfaces can be predesigned for affecting ice propagation, growth and following evolution. It should be noted that both ice growth/propagation inhibitors and programming ice‐free zone cannot make surfaces free of ice. These methods provide alternative choices in specific situations when ice‐free surfaces in confined regions and limited time are required. Dynamic ice melting using solar illumination can be a better choice for outdoor large‐scale anti‐icing applications. However, more studies on the improvement of solar absorption efficiency of these surfaces, especially with ice/snow covering, are still highly essential before these surfaces are ready for practical applications.

The DAIS also offers alternative pathways for solving the durability problem of icephobic surfaces. Generally, anti‐icing surfaces are designed by modifying the contacts between water/ice and surfaces, for instance, using micro/nanostructured surfaces for depressing water–surface interaction and designing surfaces with low surface energy for weak ice–surface interactions. Such surfaces have poor durability owing to the degradation of the surface topography in usage, because surface nanostructures and chemicals degradation are almost unavoidable in practice. The icephobicity of SAIS commonly suffers from such irreversible decay. There is an unavoidable trade‐off in the performance of SAIS, namely, choosing hard materials for the increase the durability by sacrificing of a decrease in icephobicity or using soft materials for the increase the icephobicity by sacrificing of a decrease in durability. The DAIS pinpoints the importance of evolutions after ice formation in anti‐icing surfaces design. One promising durable icephobicity could be achieved by integrating tough materials/structures with large capacity of abrasion resistance. Such tough materials/structures can provide long‐term durability, while the dynamic components in the surface can weaken ice–substrate interactions and provide excellent icephobicity. More works focus on enabling dynamic functions into robust surfaces and on extending the life spans of dynamic components can promote practical applications of the design of durable icephobic surfaces. There is still a long way for well‐established DAIS approaching large‐scale applications. First, DAIS such as stimuli‐responsive surfaces and photothermal trap surfaces integrate expensive functional components, which significantly increase the cost of large‐scale fabrication. Although alternative methods, such as utilizing PTSLIPS,^[^ ^36^ ^]^ is relatively low‐cost with abundant materials available, the scale‐up performance still needs further exploration. Furthermore, there is currently no large‐scale demonstration of DAIS available. Extensive studies focusing on scaling up fabrication will speed up transformation of DAIS from laboratory products to practical application.

Given the abundant research results on anti‐icing materials, the understanding of the icing problem also needs to be updated. Regarding ice formation on a surface, it is crucial to realize the dynamic evolution of the interaction between the ice and its substrates. For the practical usage, the surfaces that can maintain long time‐span dynamic properties and can respond to external stimuli are of promising potentials. For instance, ice adhesion on the liquid layer generators and other surfaces that secrete interfacial lubricant can constantly decrease to a negligible level with time. As such, automatic ice removal can be realized even in the outdoor environment if the functionality life‐time of such surfaces is further enhanced. Several anti‐icing surfaces have combined external energy inputs in design. It is fair to say that energy input from solar illumination is one of the most favorable and sustainable choices. Such surfaces still have relatively low power density or suffer from critical environmental limitation such as extremely low temperature, which deserves continuous research effort in the future research. Last but not least, the rational structure–property–function relationship in the current anti‐icing surfaces is still missing. It is certainly difficult to establish this relationship given that approaches to realize surface icephobicity vary greatly as well as a wide variety of materials have been selected for surface fabrication. More theoretical researches on DAIS and new approaches should be promoted, including atomistic modeling and simulation, multiscale approaches, and even machine learning. Based on the current fast evolving status of anti‐icing research, the society can hold an optimistic attitude on unwanted icing and expect practical passive dynamics anti‐icing surfaces in a very near future.

## Conflict of Interest

The authors declare no conflict of interest.
